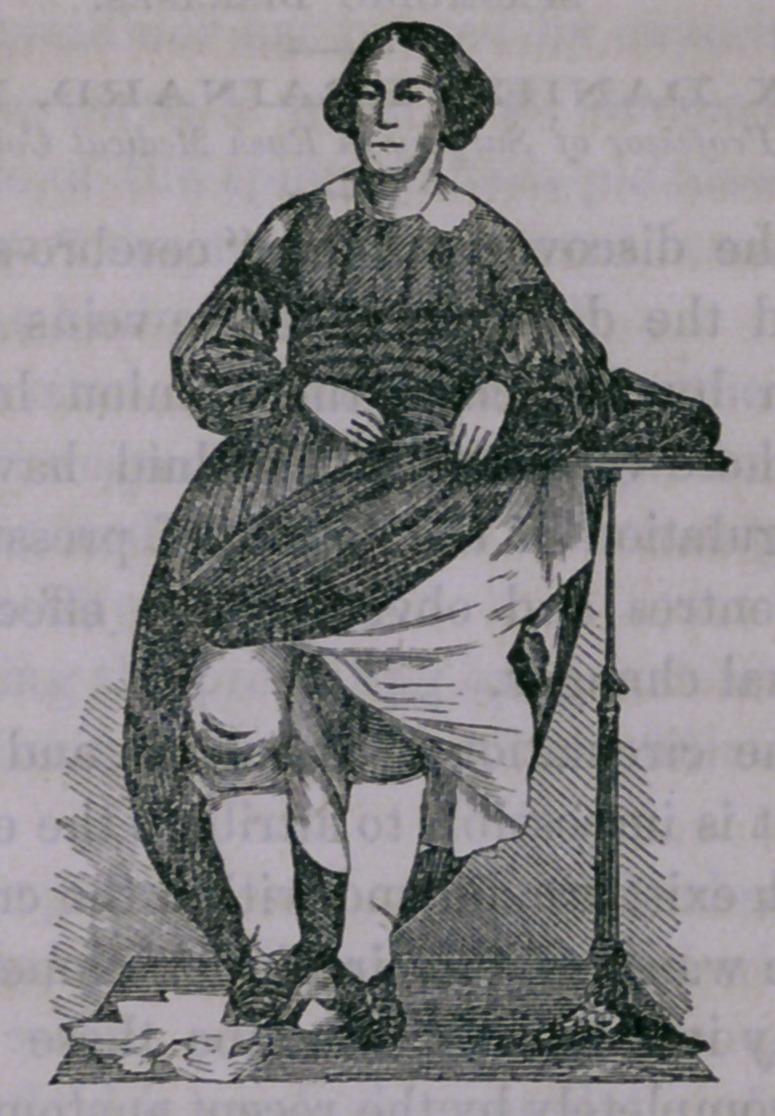# Resection of the Knee Joint

**Published:** 1861-01

**Authors:** J. W. Freer

**Affiliations:** Professor of Surgical and Microscopical Anatomy in Rush Medical College


CHICAGO
### MEDICAL JOURNAL.

VOL. IV.] JANUARY, 1861. [NO. 1.

### ORIGINAL COMMUNICATIONS.

### A STATEMENT

OF THE PRESENT CONDITION OF A CASE OF RESECTION OF THE KNEE JOINT, PERFORMED IN FEBRUARY, 1859.

BY J. W. FREER, M. D.,
*Professor of Surgical and Microscopical Anatomy in Rush Medical College.*

The July number, 1859, of the *Chicago Medical Journal*, contains the report of the above case; but for the information of those who may not be in possession of the number referred to, I will recapitulate briefly the important points.

The patient was a female, aged 30 years, of healthy and vigorous constitution. The appearances of the diseased limb were reported as follows: Joint enormously swollen, partly from pressure of fluid within the articular cavity, and partly from œdema. Spontaneous dislocation of the leg outward, with semi-flexion and anchylosis; in front and below, a depressed cicatrix, marking the situation of a former fistulous opening; the skin of natural color and temperature.

The operation was performed on the 10th of February, 1859, in the following manner: The patient being placed fully under the influence of chloroform, and in proper position, an incision was made in outline like the letter U, the inferior portion corresponding with the tibia, an inch below the lesser tuberosity, the open sides extending upward on either border of the joint, to the distance of four inches above the articulation. The flap being dissected up, the ligamentum patellæ and lateral ligaments severed, the extremities of the bones were readily exposed by forcible flexion. Having removed the patella, excision was easily completed with the amputating saw. Two inches in length of the femur and one inch of the tibia were removed.

The morbid anatomy of the joint showed an entire destruction of the articular cartilages, and portions of the bones were deeply excavated.

The treatment was that of confining the limb in a straight carved splint and foot board, with a long side splint firmly attached to the pelvis and foot. No medical treatment was required, not even anodynes, as the patient did not suffer pain enough to prevent sleep any time during her confinement in bed.

Four months after the operation, the wound had healed and the purulent discharge ceased, while union had taken place of sufficient firmness to sustain the weight of the limb without splints.

Eight months after the operation, she was able to go about with the aid of one crutch, union being sufficiently firm to sustain the weight of the body in the upright position.

At the present time—about two years since the operation—she is capable of walking without difficulty, and has thrown away crutch and cane. The limb is shortened about two and one half inches, which is compensated by means of a high-heeled shoe.

The accompanying engraving gives a very good representation of the present appearance of the limb:

I will abstain from making any remarks in regard to the propriety or impropriety of such operations, merely giving the result of the case. The subject, of late, has been sufficiently discussed by those whose experience and observation in regard to this special subject, must enable them to pronounce with some degree of certainty as to whether it is an operation fit to be performed or not. From my own investigations and observations, I am led to believe that, as far as safety to the patient is concerned, it is attended with no more, if as much, danger as in amputations above the knee. Especially where the entire constituents of the joint are removed, thus reducing the operation to the simplicity of an ordinary amputation. Where portions of synovial and cartilaginous structure are left remaining, the cure is not only protracted, but endangered, from the well-known fact that those textures have but little tendency to recover from inflammatory action, and the destructive process proceeds with characteristic tardiness.

## Figures and Tables

**Figure f1:**